# Superior Mesenteric Vein Dilatation With Ladd’s Band Causing Partial Intestinal Obstruction in a Five-Year-Old Male Child: A Case Report

**DOI:** 10.7759/cureus.45895

**Published:** 2023-09-25

**Authors:** Layth J. M. Saada, Nisa Begum Erdogan, Jamil Saada

**Affiliations:** 1 Faculty of Medicine, Al-Quds University, Jerusalem, PSE; 2 Faculty of Medicine, Istanbul University-Cerrahpasa, Istanbul, TUR; 3 Pediatric Surgery Department, Al Mezan Hospital, Hebron, PSE

**Keywords:** exploratory laparotomy, abdominal pain, vomiting, ladd’s band, superior mesenteric vein, midgut malrotation

## Abstract

Midgut malrotation has a noticeable frequency among congenital anomalies and presents mostly as vomiting. We report a case of a five-year-old boy with recurrent attacks of bilious vomiting and mild epigastric colicky abdominal pain for a year. Midgut malrotation with Ladd's band and superior mesenteric vein (SMV) dilatation causing partial intestinal obstruction were diagnosed based on the clinical presentation, upper gastrointestinal (GI) barium follow-through study, computed tomography (CT) scan with and without IV contrast, and intraoperative findings. In the upper GI barium study, the duodenum was passing anteriorly, and the second and proximal third portions were persistently dilated with recurring to-and-fro type peristalsis, resulting in delayed passage through the third portion. In the abdomen and pelvis CT scan with and without IV contrast, the SMV was dilated, forming a venous collar around the third portion of the duodenum and causing partial obstruction. Exploratory laparotomy revealed a hugely dilated SMV trapped in the Ladd’s band. Ladd’s procedure was done besides releasing the SMV and widening the root of the mesentery. The postoperative follow-up was smooth, without any relapse of the previous symptoms. Midgut malrotation in the older age group is rare in itself because there are relatively few cases documented in this age range. We determined to present this case to raise awareness of knowledge concerning the diagnosis and timely management of this condition in order to prevent comorbidity. In addition, we realise that SMV dilatation is an uncommon correlation of the known condition, midgut malrotation, and hope to contribute to the literature.

## Introduction

Intestinal malrotation is a congenital anomaly in which the midgut fails to spin entirely or partially during the early embryological developmental period, and approximately 75-85% of such patients are detected in early infancy with more specific and typical symptoms, while the minority of cases may be missed until childhood, as in our case, or perhaps even adulthood, caused by a broad variety of atypical mild small bowel obstruction symptoms [1]. The incidence of Ladd's band midgut malrotation is approximately 1:500 to 1:5000 live births [2]. Sometimes it can manifest as a potentially fatal condition with volvulus.

## Case presentation

A five-year-old boy was referred to the paediatric surgery department electively with recurrent attacks of vomiting for a year. The vomitus was moderate, yellowish-greenish, bilious, non-bloody, and non-projectile, with three episodes per day. The first episode lasted for four days and was then alleviated for a six-month symptom-free period. The second episode had the same clinical scenario that lasted for a week, then was alleviated for two months. Once there, the third episode began, repeating every month with an increasing frequency of attacks. The vomitus was associated with nausea and intermittent mild epigastric colicky abdominal pain with no radiation, provoked by eating and relieved by vomiting. There was no change in bowel or bladder habits, signs of dehydration, or a significant change in weight. There was no history of fever, cough, yellowish discoloration, headache, seizure, or blurred vision. He had a free medical and surgical history.

The patient was cooperative and oriented, with no jaundice, cyanosis, or pallor. His vital signs were within normal limits, and his recent weight was 18 kg. The abdomen was flat, with an inverted umbilicus at the midline. Bowel sounds were normal. The abdomen was soft and lax without tenderness, masses, or organomegaly. There were normal tympanic sounds on abdominal percussion.

**Figure 1:**
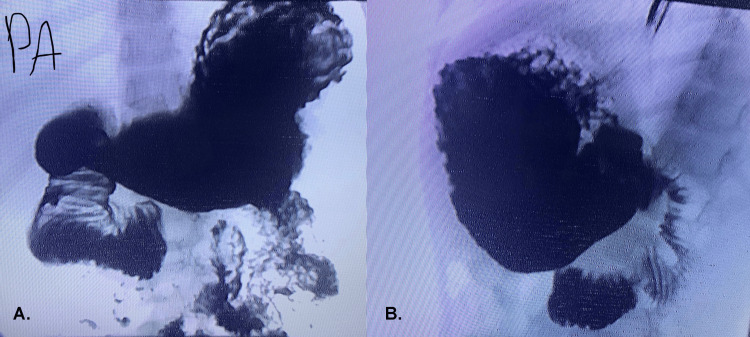
Upper gastrointestinal barium study (A) Posteroanterior view of upper GI barium study; showed persistent dilatation of the second and proximal third portions of duodenum at the level of the spine. (B) On lateral view of upper GI barium study; duodenum passes anteriorly and not inferiorly, and the DJ junction is to the left side of the spine.

Electrolytes and the complete blood count were both within normal limits. There were no significant findings on the standing abdominal X-ray. There were no notable findings other than mild distal esophagitis and unremarkable biopsy results on the upper gastrointestinal (GI) endoscopy report that the patient had. An abdominal ultrasound was performed; there are some reported findings of malrotation, but further investigations were recommended. In an upper GI barium study, at the level of the third portion of the duodenum to the right side of the spine, there was persistent dilatation of the second and proximal third portions of the duodenum with a recurrent to-and-fro type of peristalsis with delayed passage through the third portion (Figure 1A). The fourth portion ends at the ligament of Treitz. On the lateral view, the duodenum passes anteriorly and not inferiorly, and the duodeno-jejunal (DJ) junction is to the left side of the spine (Figure 1B). These findings can be those of superior mesenteric artery syndrome (SMAS) or Ladd’s band.

There was a high suspicion of SMAS; therefore, an abdomen and a pelvis computerised tomography (CT) scan with and without IV contrast were performed. This revealed an ectopic left kidney in the pelvis with anterior malrotation and a dilated extra-renal pelvis, superior mesenteric vein (SMV) dilatation forming a venous collar around the third portion of the duodenum, which was superiorly compressed by the portal vein, an abnormal course and ring-like formation around the proximal jejunum causing partial obstruction, and the bowel - due to non-optimal filling - looks normal without evidence of wall thickening or dilatation.

**Figure 2:**
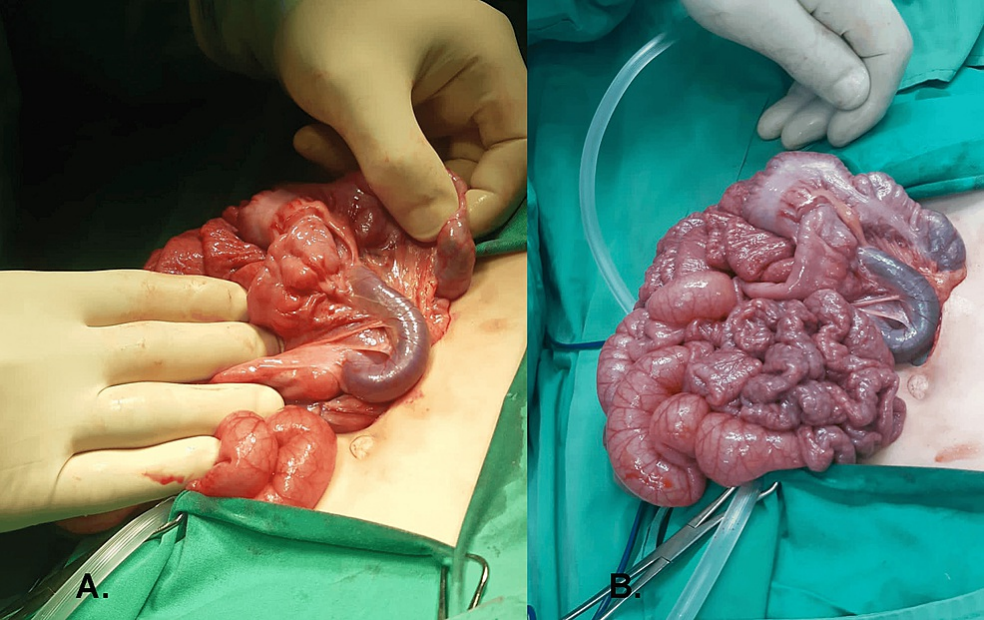
Superior mesenteric vein and collapsed small bowel (A) Significantly dilated superior mesenteric vein and (B) collapsed small bowel distal to the ligament of Treitz.

Exploratory laparotomy was done and revealed a narrow mesenteric root, a cecum in the mid-upper abdomen with Ladd’s band crossing the third portion of the duodenum, a significantly dilated superior mesenteric vein whose diameter was around 1.5 cm, which was trapped in these bands (Figure 2A), and a collapsed small bowel distal to the ligament of Treitz (Figure 2B).

**Figure 3:**
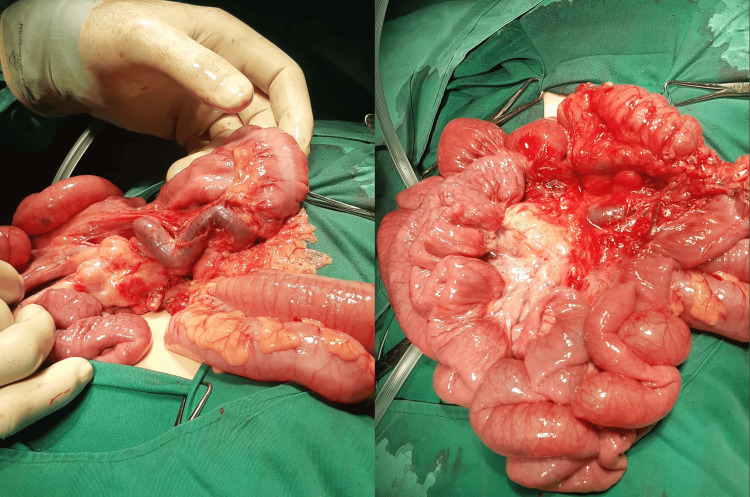
Releasing the superior mesenteric vein Showed division of the Ladd’s band, releasing the superior mesenteric vein, and widening the root of mesentery.

Ladd’s procedure, along with appendectomy, was done, the superior mesenteric vein was released, and the root of mesentery was widened (Figure 3).

The postoperative period passed uneventfully, and the patient was discharged on the third day after resuming oral feeding and passing normal bowel motions. The follow-up during the next five months was smooth, without any relapse.

## Discussion

Midgut malrotation is a congenital anomaly of fixation, internal rotation, and the bowel failing to rotate a total of 270° counterclockwise around the axis of the superior mesenteric artery (SMA) [3]. Midgut malrotation is a newborn disease because it typically manifests as a clinical emergency related to malrotation, such as volvulus, or as duodenal obstruction symptoms in the first month of life. Although 75-85% of such patients are detected with more specific symptoms in early infancy, a minority of cases, such as ours, may go missing until childhood due to chronic and atypically mild small intestinal obstruction symptoms [1]. Patients in childhood and adolescence are more likely to present with frequent abdominal pain, recurrent episodes of intestinal obstructive symptoms, or failure to thrive due to intestinal obstruction or intestinal ischemia [4,5]. In our case, the child presented with recurrent attacks of bilious vomiting associated with nausea and mild epigastric colicky abdominal pain for a year with no significant change in weight.

Intestinal malrotation can be present with a midgut volvulus accompanied by ischemia and necrosis, which is mostly associated with a pathognomic pattern called the "whirlpool sign," which corresponds to a clockwise wrapping of the SMV and the mesentery around the SMA. The SMV can be found to be collapsed or dilated. Pracros et al. examined six patients with Ladd’s band and without volvulus, none of whom presented the "whirlpool sign" in ultrasound imaging [6]. In our case, there were no volvulus signs on ultrasound imaging, and the whirlpool sign was not observed.

According to Stringer’s classification, there are three main types of midgut malrotations based on the embryological state of development: type 1 non-rotation, in which the DJ junction lies on the right and the colon on the left; type 2 incomplete rotation, also known as duodenal malrotation, in which the cecum in the epigastric region overlies the third part of the duodenum; and type 3 reverse rotation, also known as combined duodenal and cecal malrotation, in which the DJ loop is anterior to SMA and the transverse colon is posterior to SMA [7]. An upper GI barium study and follow-through should be performed in all events of bilious vomiting, as well as in patients with chronic vomiting, to rule out malrotation [8].

The upper GI barium study in our case showed a duodenum passing anteriorly, a DJ junction on the left side of the spine, the fourth portion of the duodenum ending at the ligament of Treitz, and a cecum in the mid-upper abdomen with Ladd’s band crossing the third portion. Hence, it seems that it contains features of both type 2 'incomplete rotation' and type 3 'reverse rotation'. It also showed findings corresponding with the SMAS, a cause of vomiting resulting from the compression of the third part of the duodenum between the SMA and the aorta [9]. After performing an abdomen and pelvis CT scan with and without IV contrast, narrowing of the aortomesenteric angle was not found, and SMAS was ruled out.

Laparoscopic or open Ladd’s procedure is the standard surgical management for intestinal malrotation [10]. Timely diagnosis and management improve the quality of life for these children.

We remark that SMV dilatation is a rare association of the known disease, midgut malrotation, and hope to contribute to the literature.

## Conclusions

Since there are few reported cases of midgut malrotation in this age range, we determined to present this case to raise awareness of knowledge concerning the diagnosis and timely management of this condition in order to prevent comorbidity. Furthermore, we recognise that SMV dilatation is a rare association with the well-known condition, midgut malrotation, and we hope to contribute to the literature.
